# Oral Phenotype and Salivary Microbiome of Individuals With Papillon–Lefèvre Syndrome

**DOI:** 10.3389/fcimb.2021.720790

**Published:** 2021-08-26

**Authors:** Giulia Melo Lettieri, Luander Medrado Santiago, Giancarlo Crosara Lettieri, Luiz Gustavo dos Anjos Borges, Letícia Marconatto, Laudimar Alves de Oliveira, Nailê Damé-Teixeira, Loise Pedrosa Salles

**Affiliations:** ^1^Department of Dentistry, Faculty of Health Sciences, University of Brasilia, Brasília, Brazil; ^2^Periodontology Research Group, Specialized Center in Periodontology and Implantology, Brasília, Brazil; ^3^Microbial Interactions and Processes Research Group, Helmholtz Centre for Infection Research, Braunschweig, Germany; ^4^Institute of Petroleum and Natural Resources, Pontifical Catholic University of Rio Grande do Sul, Porto Alegre, Brazil

**Keywords:** Papillon–Lefèvre disease, cathepsin C, periodontal infection, saliva, microbiology, periodontitis

## Abstract

Papillon–Lefèvre syndrome (PLS) is an autosomal recessive rare disease, main characteristics of which include palmoplantar hyperkeratosis and premature edentulism due to advanced periodontitis (formerly aggressive periodontitis). This study aimed to characterize the oral phenotype, including salivary parameters, and the salivary microbiome of three PLS sisters, comparatively. Two sisters were toothless (PLSTL1 and PLSTL2), and one sister had most of the teeth in the oral cavity (PLST). Total DNA was extracted from the unstimulated saliva, and the amplicon sequencing of the 16S rRNA gene fragment was performed in an Ion PGM platform. The amplicon sequence variants (ASVs) were obtained using the DADA2 pipeline, and the taxonomy was assigned using the SILVA v.138. The main phenotypic characteristics of PLS were bone loss and premature loss of primary and permanent dentition. The PLST sister presented advanced periodontitis with gingival bleeding and suppuration, corresponding to the advanced periodontitis as a manifestation of systemic disease, stage IV, grade C. All three PLS sisters presented hyposalivation as a possible secondary outcome of the syndrome. Interestingly, PLST salivary microbiota was dominated by the uncultured bacteria *Bacterioidales* (F0058), *Fusobacterium*, *Treponema*, and *Sulfophobococcus* (*Archaea* domain). *Streptococcus*, *Haemophilus*, and *Caldivirga* (*Archaea*) dominated the microbiome of the PLSTL1 sister, while the PLSTL2 had higher abundances of *Lactobacillus* and *Porphyromonas*. This study was the first to show a high abundance of organisms belonging to the *Archaea* domain comprising a core microbiome in human saliva. In conclusion, a PLST individual does have a microbiota different from that of the periodontitis’ aggressiveness previously recognized. Due to an ineffective cathepsin C, the impairment of neutrophils probably provided a favorable environment for the PLS microbiome. The interactions of *Bacteroidales* F0058, *Caldivirga*, and *Sulfophobococcus* with the microbial consortium of PLS deserves future investigation. Traditional periodontal therapy is not efficient in PLS patients. Unraveling the PLS microbiome is essential in searching for appropriate treatment and avoiding early tooth loss.

## Introduction

Papillon and Lefèvre first described the Papillon–Lefèvre syndrome (PLS) in 1924 (Papillon and Lefevre, 1924). PLS is a hereditary autosomal recessive and rare condition that affects one to four people per million (Hart and Shapira, 1994). Generally, consanguineous marriages are PLS individuals’ origin (Bhavsar et al., 2013; Bullón et al., 2018). It was estimated that over one billion people live in countries where consanguineous marriages are customary (Hamamy et al., 2011). Among them, one in every three marriages is between cousins. The main impact of consanguinity is the increased expression of multiple mutations encoding rare autosomal recessive genetic disorders, with an increased risk for first cousin couples to bear affected children. The PLS’s main phenotypic characteristics are palmoplantar hyperkeratosis and the premature loss of deciduous and permanent teeth. The edentulism process in PLS starts with aggressive periodontitis (AIBarrak et al., 2016). In the period of dental exfoliation, the gingival tissue of PLS patients becomes hyperplastic and hemorrhagic, and there is an extended significant bone loss of the maxilla and mandible, cement exposure, and tooth mobility that culminates in loss of teeth (Robertson et al., 2001; Jordan, 2004; Tumen et al., 2015). Therefore, poor quality of life is expected in childhood, as it corresponds with the most destructive period of the disease. The periodontal condition and tooth loss generate high sensitivity in patients with PLS and poor diet quality (Hattab, 2019). These subjects present an increased incidence of skin and oral infections, which led to a substantial immunological disorder hypothesis at the first line of cellular defense. The immune system impairment may explain the predisposition to oral infection and periodontitis in PLS as a primary etiological component.

The PLS results from mutations in the cathepsin C gene (*CTSC*), also known as dipeptidyl peptidase 1 (*DPPI*), located on chromosome 11q14. The *CTSC* mutations produce an inactive cysteine protease or reduce its function (Fischer et al., 1997; Hart et al., 1999; Toomes et al., 1999). Currently, 113 *CTSC* variants have been reported in ethnically various populations, including a novel missense variant in exon 6 of the cathepsin C gene of a PLS Chinese individual (Yu et al., 2021). Over 90% of the variants were missense variants, nonsense variants, or frameshift variants, and most of them were in exons 5–7 of *CTSC*. Cathepsin C is an essential lysosomal enzyme in the cascade of activation of immune and inflammatory cell serine proteases and other cell lineages. The majority of proteins that demand cathepsin C processing are essential for the innate immune system’s proper function. Neutrophil elastase, proteinase-3, and granzymes A, B, and C are examples of proteins that depend on cathepsin C-mediated cleavage for activation (Kaplan and Radic, 2012). Bullón et al. (2018) showed autophagosome accumulation in mutant fibroblasts from a PLS patient’s skin. The autophagosome accumulation was associated with alterations in oxidative/antioxidative status, reduced oxygen consumption, and a marked autophagic dysfunction. Immune and inflammatory cells also showed dysfunctional behavior in PLS. For instance, neutrophils demonstrated hyperactivity with increased oxidative stress and reduced capacity to form neutrophils’ extracellular trap structures (NETs) (Sørensen et al., 2014; Roberts et al., 2016). NETs are important defensive structures composed of DNA, chromatin, and bactericidal proteins (Kaplan and Radic, 2012). Sørensen et al. (2014) showed that PLS patients neutrophils lack or had significantly reduced amounts of neutrophils elastase (NE), cathepsin G (CTSG), proteinase 3 (PR3), and azurocidin (CAP37). Azurocidin is a member of the neutrophil serine proteases, with intense chemotactic activity toward monocytes, and the formation of NETs depends on the presence of NE. Excessive or diminished NET production may lead to autoimmune and inflammatory disorders, like inflammasome activation, interfering significantly in PLS patients’ defensive mechanisms. Scientific reports of CTSC^−/−^ mice show that neutrophil granulocyte is altered in the absence of CTSC (John et al., 2019). Moreover, the neutrophil serine protease elastase (NE) activity was markedly reduced by approximately 50% in the knockout granulocytes. Other neutrophil serine proteases, cathepsin G (CTSG) and proteinase 3 (PR3), were strongly reduced as well. The cleavage of the cell–cell contact molecule E-cadherin was also impaired in the absence of CTSC, suggesting that the impaired tissue infiltration of CTSC^−/−^ neutrophils are caused by reduced E-cadherin cleavage at adherens junctions rather than by reduced motility of neutrophils (John et al., 2019). Taken together, the mutations in the cathepsin-C gene and the immune/inflammatory cell dysfunction may explain PLS patients being more prone to oral dysbiosis and proliferation of periodontal biofilms. More critical, PLS individuals may present a diverse biofilm challenging to control with the conventional treatment.

The oral microbiota in dysbiosis is a relevant factor in several oral conditions such as dental caries, periodontitis (PD), apical lesions, alveolar osteitis, and tonsillitis. It also plays a role in certain systemic diseases such as cardiovascular disease, diabetes mellitus, pneumonia, and premature births (Dewhirst et al., 2010). Nonetheless, the knowledge of the microbial community is essential to evaluate the effects of these microorganisms in the host and might help the evolution of treatments for oral disorders (Socransky and Haffajee, 2005). However, the oral microbiome in PLS is not fully described or related to the phenotype of the syndrome. This knowledge gap might explain why the periodontitis (PD) treatment is not efficient in those patients (Albandar et al., 2012). The hypothesis for the unsuccessful PD therapy in PLS is the combination of the existence of pathogenic species, the onset inflammation (Kilian et al., 2016; Hoare et al., 2018), and the immune disturbance caused by cathepsin C mutation that is not well understood (Albandar et al., 2012; Kilian et al., 2016). Therefore, understanding the triad “oral microbiota, immune/inflammatory response, and cathepsin-C mutation” is mandatory to search for a successful periodontal and systemic treatment of PLS, currently unavailable. This study aimed to characterize the oral phenotype, including salivary parameters, and the salivary microbiome of three PLS sisters, comparatively. Two of them were already edentulous, while the younger one had 15 teeth in the oral cavity. To achieve our purpose, we chose a combination of universal primer pairs for the 16S rRNA gene with suitable putative coverage for archaea and the analysis of the amplicon sequence variants (ASVs) followed by taxonomy assignment using the SILVA v.138. Sequence variations in 16S and other metagenomic loci contain phylogenetic information that can be used to infer the taxonomic relationships of the microbial hosts (Fricker et al., 2019). Analysis of ASVs provides improved sensitivity and specificity and reduces the problem of inflated microbiota datasets due to falsely identified distinct operational taxonomic units (OTUs) originating from misclustered sequences (Callahan et al., 2017). According to the authors, the ultimate reference-free statistical denoising methods such as Dada2 overcome the non-reproducibility of OTU clustering results with modified or expanded datasets by recovering independent biological sequences as ASVs, promoting reproducibility and comparability of amplicon-based microbiome analysis.

## Materials and Methods

### Participants and Clinical and Radiographic Examination

This research was approved by the Research Ethics Committee of the School of Health Sciences of the University of Brasília (FS-UnB; no. 2.974.167) and performed under the World Medical Association (WMA) Declaration of Helsinki. Individuals were informed verbally about the study’s objective and signed a consent form. Three sisters from a consanguineous marriage of first cousin couples and diagnosed with PLS were selected for this study. Characteristics preintervention included advanced periodontitis, palmoplantar hyperkeratosis, and a long story of failed dental treatment. The sisters were submitted to clinical, photographic, and radiographic examinations. Two PLS sisters had lost all their teeth approximately 2 years before the study enrolment, except for the impacted third molars: Papillon–Lefèvre syndrome toothless 1 (PLSTL1) and Papillon–Lefèvre syndrome, toothless 2 (PLSTL2). One sister had 15 teeth in her mouth [Papillon–Lefèvre syndrome toothed (PLST)]. The sisters were 14, 16, and 18 years old at the baseline on August 3, 2018, non-smokers, and had no other systemic diseases apart from PLS. After the saliva samples collection, the PLST patient was submitted to oral hygiene instruction and motivation. An experienced periodontist treated the PLST patient with subgingival scaling and root planning under local anesthesia. The probing depth technique consisted of 4 points measurements to detect the most profound penetration areas. The disease classification was established following the 2017 World Workshop of Periodontal and Peri-implant Disease and Conditions (Caton et al., 2018). Re-evaluations were carried out 1 month later and every 3 months after the study enrolment.

### Salivary Characteristics

#### Salivary Flow and Reliability Assays

Saliva samples were collected in the morning on of August 3, 2018. Patients were asked to refrain from eating and drinking for 2 h before the sampling. None of the sisters were using systemic antibiotics or local antimicrobials or submitted to periodontal treatment for nine months previously to the saliva collection. The unstimulated salivary flowrate was performed by passive drooling, with patients seated. The collection was carried out for 5 min. The volume of saliva was measured (mL/min). The saliva reliability was analyzed during the transfer of saliva to a microtube. The stimulated salivary flow was performed by 1 min chewing a rubber dam (Madeitex, São José dos Campos, SP, Brazil). The saliva during the stimulation was discarded, and then, the flow was measured by 5 min (Navazesh and Kumar, 2008). For the phenotypic analysis, the PLS salivary samples were collected in three different sessions, with intervals of approximately 1 year. No medication or periodontal treatment was taking place 3 months before or during the saliva collection period. The classifications regarding resting salivary flow were assialia (0.00 mL/min), hyposialia (0.1–0.29 mL/min), and ideal (0.3–0.4 mL/min). Regarding stimulated salivary flow, the classifications were asialia (0.00 mL/min), severe hyposalivation (0.1–0.4 mL/min), moderate hyposalivation (0.5–0.9 mL/min), mild hyposalivation (1.0–1.4 mL/min), ideal salivation (1.5–2.5 mL/min), and sialorrhea (>2.5 mL/min). Regarding the reliability, the classifications were serous (does not form a string), fluid (a string of 2 cm), or viscous (a string of ≥5 cm).

#### Salivary pH and Buffering Capacity

A volume of 1 mL of stimulated saliva was transferred to 1.5 mL tubes, and the pH was measured using the tape method (pH-Fix 0-14, Macherey-Nagel, Düren, NRW, Germany) by 30 s. Another 1 mL of stimulated saliva was added to 3 ml of hydrochloric acid PA 37% (0.005M) (Dinâmica, Indaiatuba, Brazil) to evaluate the buffer capacity and the pH measured again after 2 min (mColorpHast MilliporeSigma, Burlington, MA, USA).

#### Salivary Glucose

The salivary glucose was analyzed using the Glucose Liquiform kit (Labtest Diagnóstica, Lagoa Santa, MG, Brazil). The glucose stabilizer Glistab (Labtest Diagnóstica, Lagoa Santa, MG, Brazil) was added to the unstimulated saliva in the proportion of 30 µL for each 3 mL of saliva. After that, the samples were centrifuged for 1 min, and 150 μL of the supernatant was mixed to 500 μL of the reagent 1 (phosphate buffer, 30 mmol/L, pH 7.5; phenol, ≥1 mmoL/L; glucose oxidase, ≥12,500 U/L; peroxidases, ≥800 U/L; 4-aminoanthypyrine, ≥290 μmol/L; sodium azide, 7.5 mmol/L; and surfactants). The samples were incubated at 37°C for 10 min, and the absorbance was measured at 505 nm using the spectrophotometer Spectramax (Molecular Devices LCC, San Jose, CA, USA). The capillary glucose (mg/dL) was evaluated for comparative purposes with Accu-Check and test strips (Roche, Basel, BS, Swiss).

#### Salivary Amylase

This assay was performed using an adapted protocol of amylase test (Labtest Diagnóstica, Lagoa Santa, MG, Brazil). The saliva samples were centrifuged for 1 min to 20,000 rpm, and the supernatant diluted 300 times with 0.85% NaCl. After this, 2 µl of the prepared saliva samples was added into 50 µl of substrate 1 (0.4 g/L starch, pH 7.0; phosphate buffer; and stabilizer), previously incubated at 37°C for 2 min, and incubated for additional 7 min and 30 s at 37°C. Then, 50 µL of the color reagent (potassium iodate, 16.7 mmol/L; potassium iodide, 271 mmol/L; and hydrochloric acid, 112 mmol/L) and 400 µL of distilled water were added, and after 5 min at room temperature, the absorbance was measured at 660 nm using the spectrophotometer Spectramax (Molecular Devices LCC, San Jose, CA, USA).

### Salivary DNA Extraction, Amplicon Sequencing, and Bioinformatics

The DNA was extracted using phenyl-chloroform as successfully described by Smalla et al. (1993) for prokaryotes cells. After DNA extraction, PCR was performed for partial amplification of 16S rRNA gene using universal primers 515F (5′-GTGCCAGCMGCCGCGGTAA-3′) and 806R (5′-GGACTACVSGGGTATCTAAT-3′) (Bates et al., 2011). The PCR conditions were as follows: 50 μL mixture, consisting of 1.5 mM MgCl_2_, 0.2 μM of each primer, 0.2 mM of each dNTP, 1 U Platinum Taq DNA polymerase, 1× PCR reaction buffer, and approximately 10 ng of genomic DNA. The PCR cycles were one initial denaturation step at 95°C/3 min, 25 cycles including denaturation at 95°C/30s, annealing at 52°C/1 min, and extension at 72°C/1min plus one final extension step at 72°C/7min. The PCR amplicons were purified using Agencount AMPure Beads (Beckman Coulter, Indianapolis, IN, USA). Library preparation was performed as described in the Ion Plus Fragment Library from an initial amount of 100 ng of DNA and sequenced at Ion PGM System (Thermo Fisher, Waltham, MA, USA) using an Ion 316 chip, following the manufacturer’s instructions. The raw dataset is deposited at the National Biotechnology Information Center (NCBI) under the BioProject PRJNA558499. The 16S rRNA gene reads were submitted to the DADA2 version 1.18 (Callahan et al., 2016) in R version 3.6.3 (R Core Team, 2021) to obtain the amplicon sequence variants (ASVs). Reads were filtered, retrieving reads longer than 100 bp and allowing a maximum of two errors per read. The error rates were estimated, and reads were dereplicated to remove redundancy. Chimeras were removed, and taxonomy was assigned using the Silva v.138 databases (Quast et al., 2013). ASVs assigned to eukaryote, chloroplast, or mitochondria were removed using phyloseq (McMurdie and Holmes, 2013) before further analysis.

## Results

### Oral Phenotype

**Figure 1:**
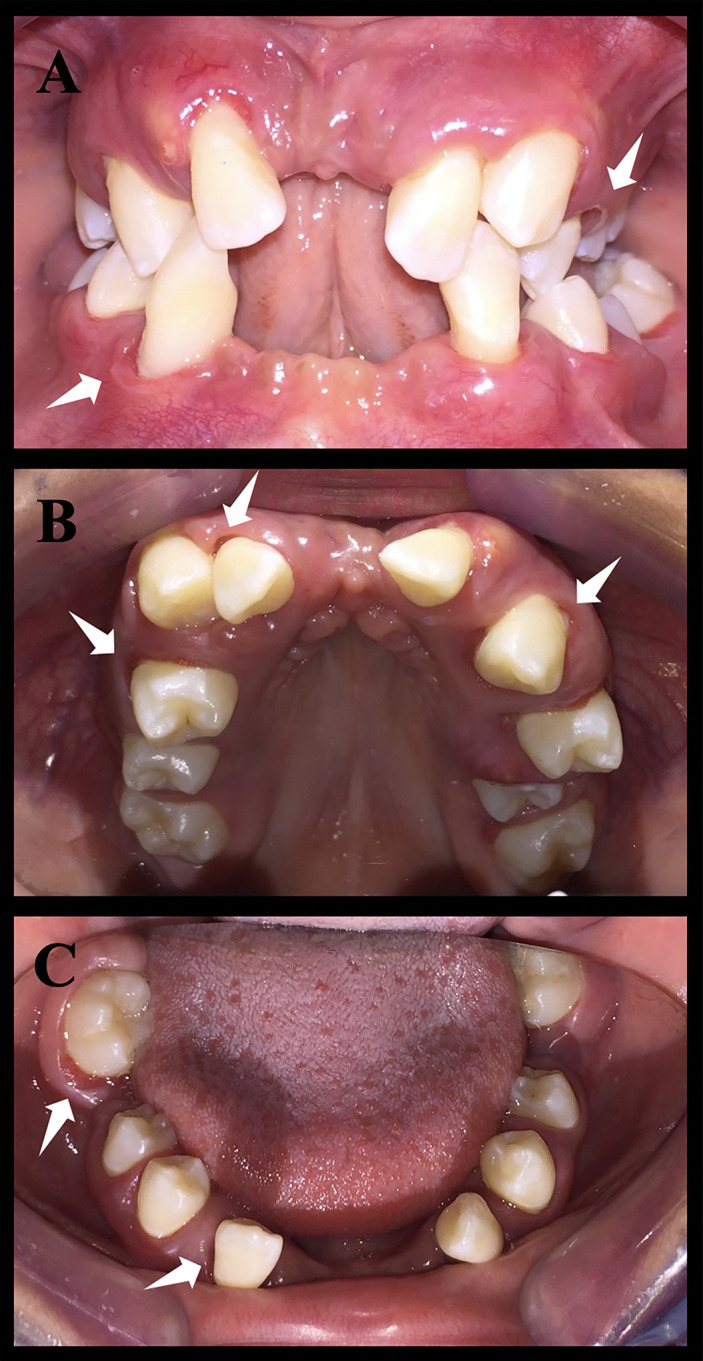
Intraoral photos of patient Papillon–Lefèvre syndrome toothed (PLST). The white arrows indicate the periodontal pockets. It is possible to notice a significant loss of dental elements. **(A)** Occlusion photo of the patient. **(B)** Upper arch photo. **(C)** Lower arch photo.

**Figure 2:**
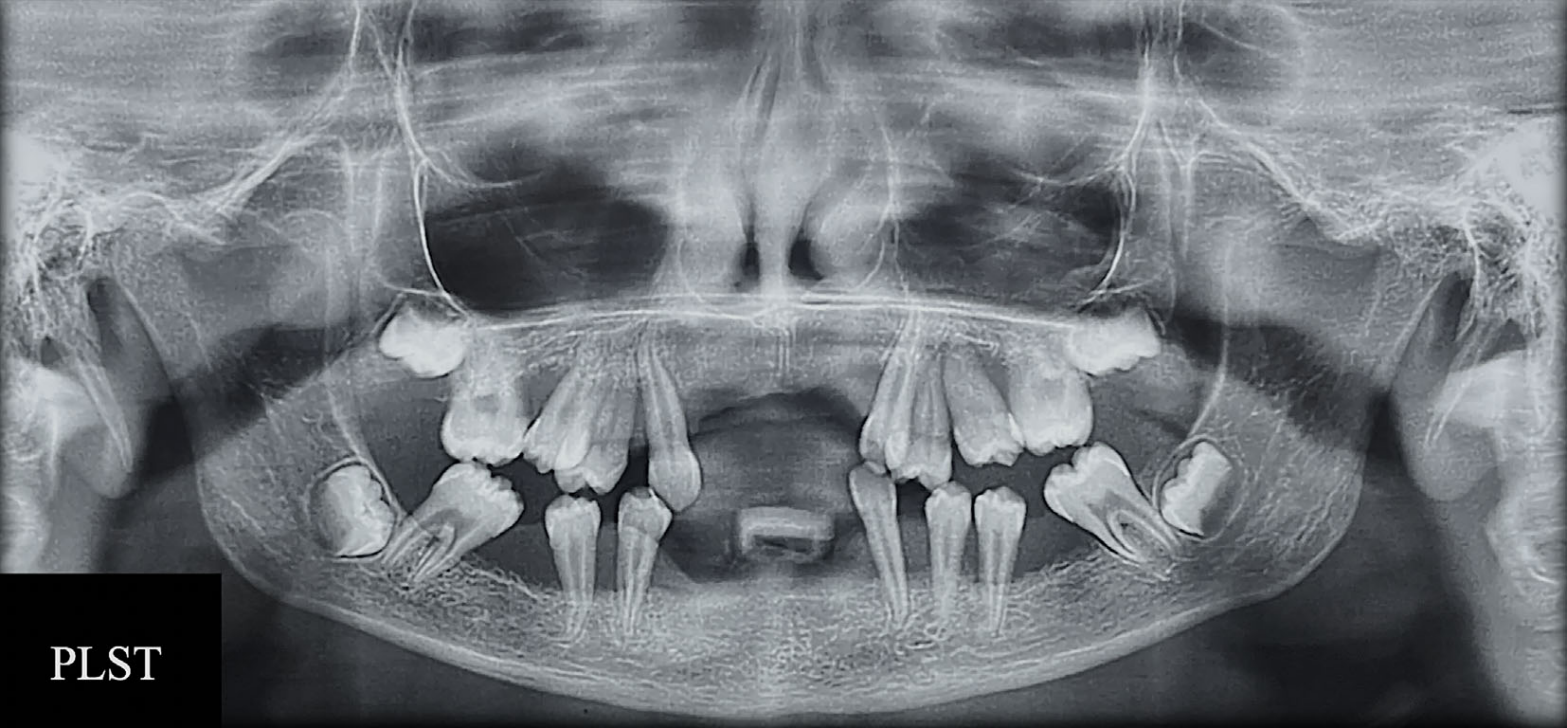
RX exam of patient Papillon–Lefèvre syndrome toothed (PLST) showing bone loss and the appearance of teeth “floating”.

In general, the phenotypic characteristics observed in the three patients were bone loss and the early loss of primary and permanent dentition, particularly for the PLSTL sisters (PLSTL1 and PLSTL2). The clinical examination of the patient who still had teeth (PLST) revealed severe gingival inflammation and hyperplasia (enlargement), spontaneous bleeding, tooth mobility, heavy dental calculus accumulation, and deep periodontal pockets (**Figures 1A–C**). The mean of PLST probing pocket depth was 8.82 mm ( ± 3.46), which did not regress with previous regular periodontal treatment (**Table 1**). The panoramic X-ray of the PLST sister showed loss of the permanent incisors and first molars. The remaining teeth had normal anatomy; however, they presented a “floating” appearance because of periodontal ligament and bone loss (**Figure 2**). The parents reported that the first symptoms for all sisters started in the primary dentition with teeth mobility and gingival bleeding. According to the new classification, the PLST condition was periodontitis as a manifestation of systemic disease (ICD-10 Q82.8), stage IV, generalized, and grade C.

**Table 1:** Baseline characteristics of the PLS individuals and salivary parameters, pH, buffering capacity, reliability, salivary flow, amylase activity, and glucose in Papillon–Lefèvre syndrome.

Individual	PLST	PLSTL1	PLSTL2
**Gender**	Female	Female	Female
**Age (years)**	14	18	16
**Number of erupted teeth**	15	Edentulous	Edentulous
**Probing Depth (mm)**	8.82 ( ± 3.46)	–	–
**Gingival Index**	2.76 ( ± 0.30)	–	–
**Unstimulated Saliva (mL/min)**	0.35 ( ± 0.17)	0.21 ( ± 0.05)	0.26 ( ± 0.06)
**Classification**	Ideal	Hypossialia	Hypossialia
**Stimulated Saliva (mL/min)**	0.73 ( ± 0.34)	0.51 ( ± 0.36)	0.26 ( ± 0.06)
**Classification**	Moderate Hyposalivation	Moderate Hyposalivation	Severe Hyposalivation
**pH**	6.97 ( ± 0.21)	7.1 ( ± 0.17)	6.7 ( ± 0.57)
**Buffering Capacity**	4.67 ( ± 1.15)	4.0 ( ± 1.0)	4.67 ( ± 1.15)
**Reliability**	Serous	Fluid	Fluid
**Glucose (mg/dL)**	1.79 ( ± 2.50)	10.11 ( ± 2.35)	1.26 ( ± 1.99)
**Amylase (U/dL)**	11,278.19 ( ± 2,040.13)	114,008.15 ( ± 2,422.31)	16,016.74 ( ± 2,399.40)
**Capillary Glucose (mg/dL)**	96	106	92

### Salivary Characteristics

**Table 1** shows the systemic and the salivary characteristics of the three PLS sisters: pH, buffering capacity, amylase activity, and glucose. The patients with PLS presented hyposialia at an unstimulated salivary flow. The stimulated salivary flow resulted in severe or moderate hyposalivation. The pH was 6.97 ± 0.21 for PLST, 7.1 ± 0.17 for PLSTL1, and 6.7 ± 0.57 for PLSTL2. The PLST salivary glycemia was 1.79 ± 2.50 mg/dL, that of PLSTL1 was 10.11 ± 2.36 mg/dL, and that of PLSTL2 was 1.26 ± 1.99 mg/dL. The PLST amylase was 11,278.19 ± 2,040.13 U/dL, while that of PLSTL1 was 114,008.15 ± 2,422 U/dL and PLSTL2 was 16,016.74 ± 2,399.40U/dL **(** **Table 1**).

### Salivary Microbiome

**Figure 3:**
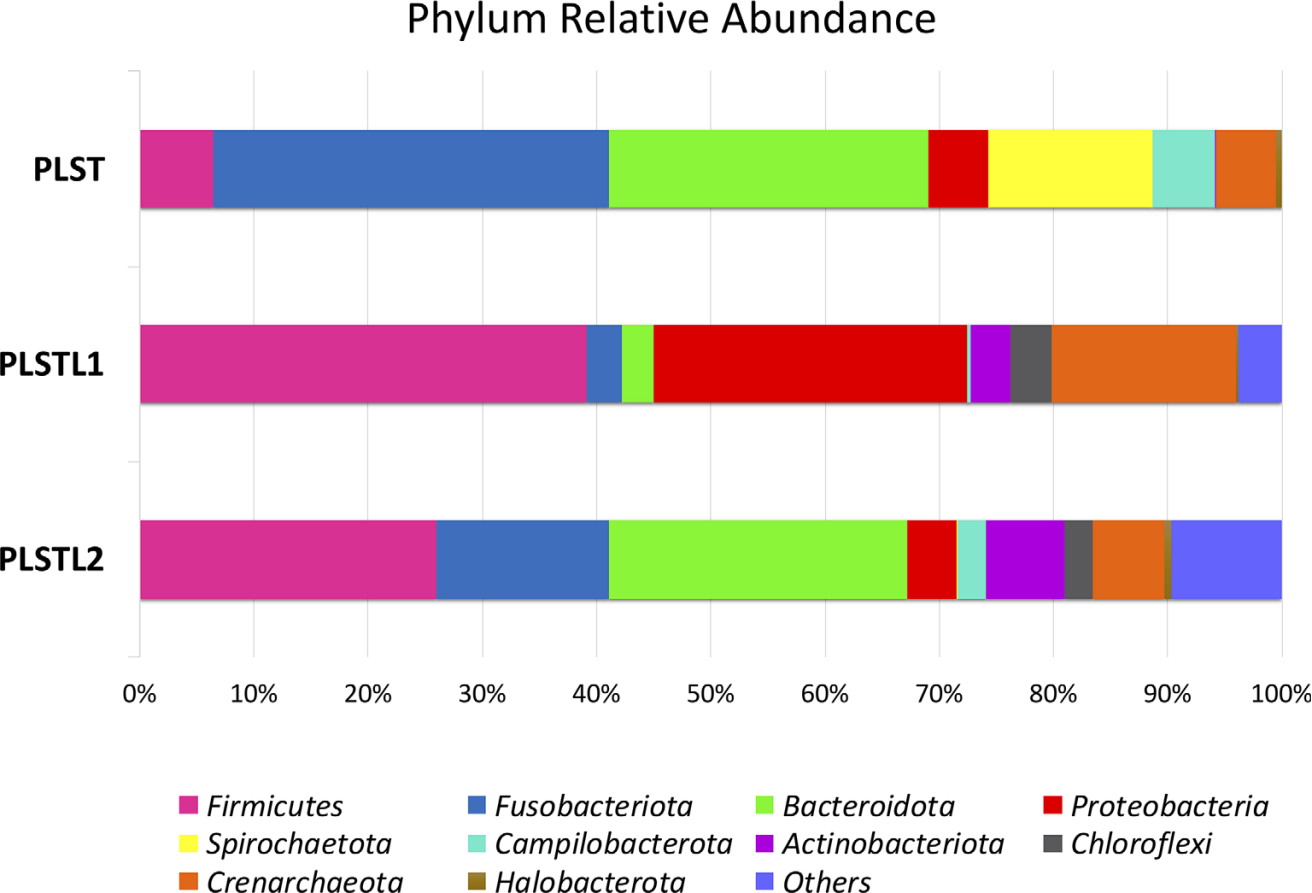
Distribution of phyla in the salivary microbiome of the sisters with Papillon–Lefèvre syndrome. Papillon–Lefèvre syndrome toothed (PLST), Papillon–Lefèvre syndrome toothless 1 (PLSTL1), and Papillon–Lefèvre syndrome toothless 2 (PLSTL2).

**Figure 4:**
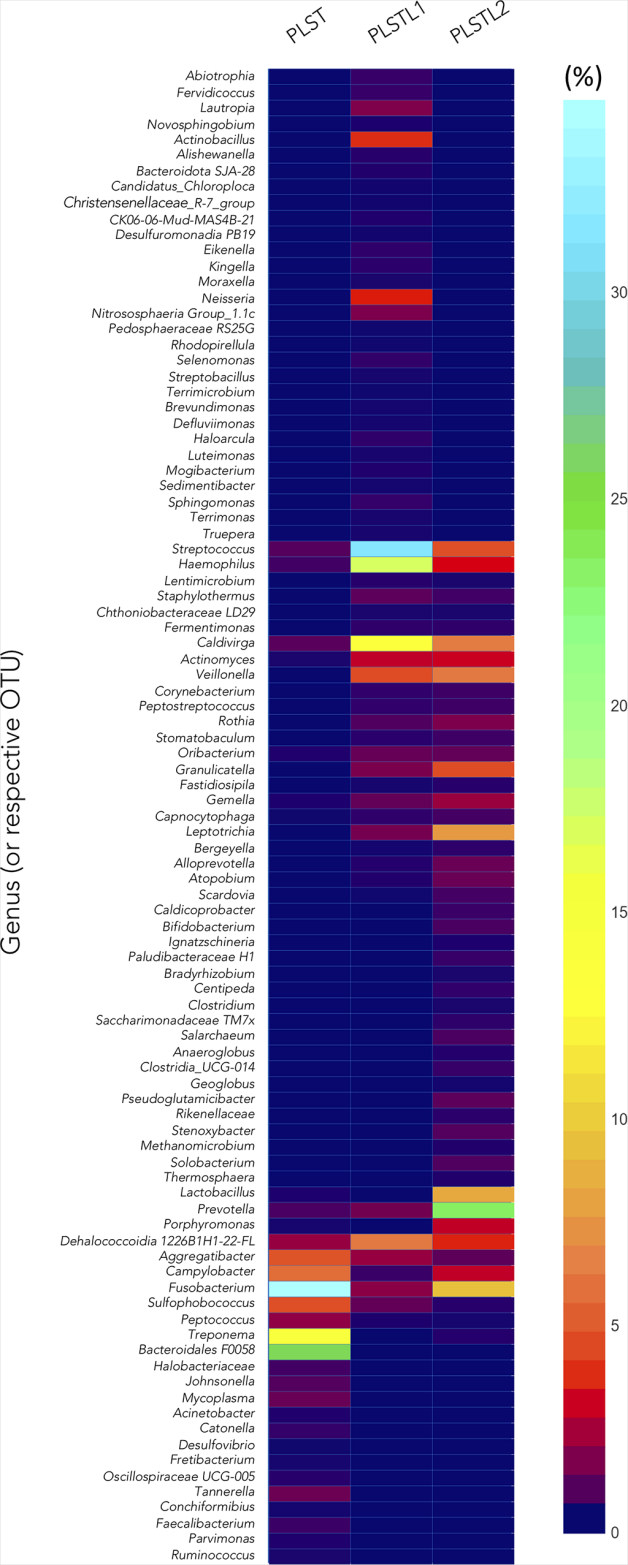
Heatmap comparing taxa composition of saliva of PLST patients. Papillon–Lefèvre syndrome toothed (PLST), Papillon–Lefèvre syndrome toothless 1 (PLSTL1), and Papillon–Lefèvre syndrome toothless 2 (PLSTL2).

**Figure 5:**
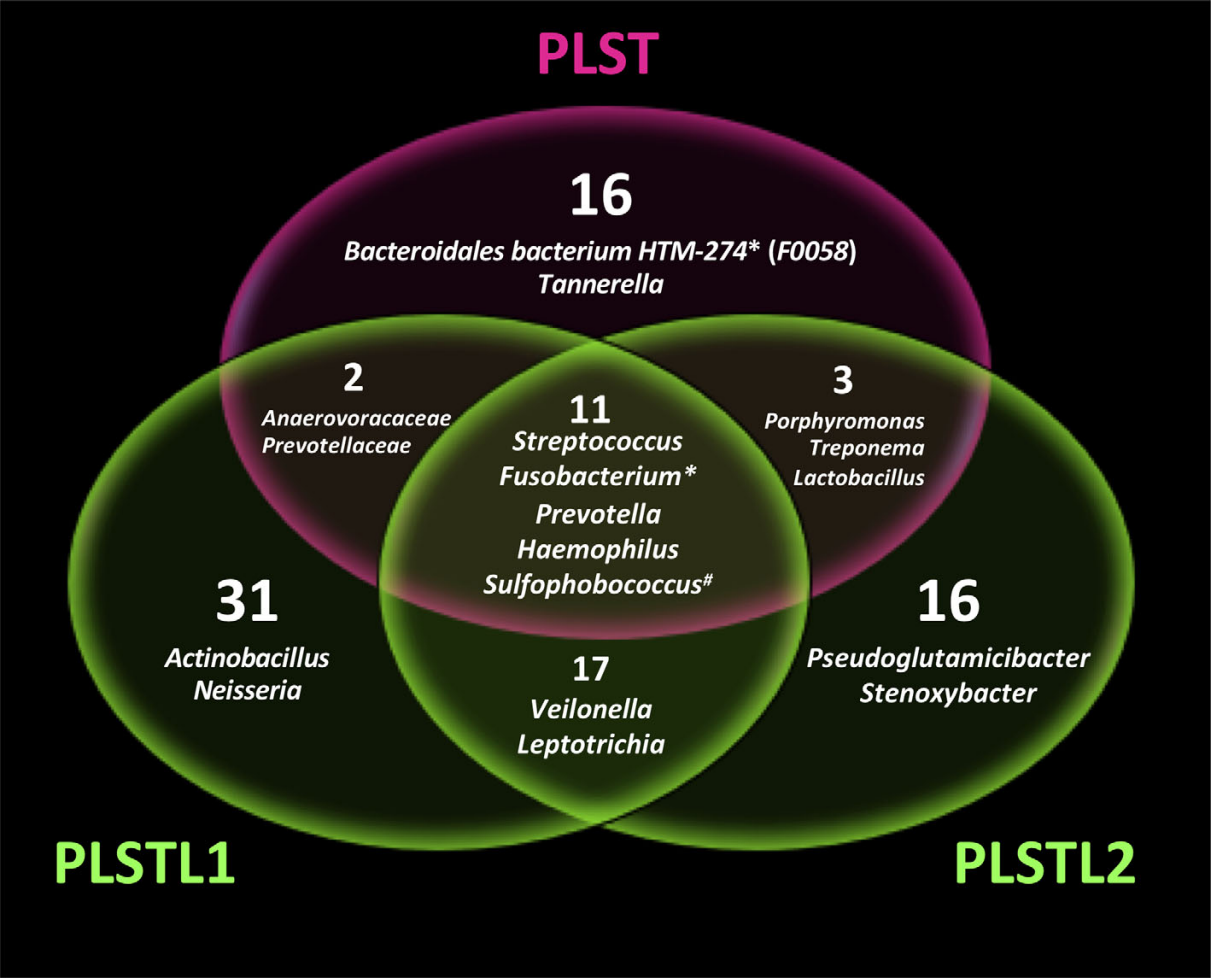
Venn diagram showing the shared microbial genus in Papillon–Lefèvre syndrome toothed patient (PLST) and toothless (PLSTL1 and PLSTL2) sisters. Asterisk indicates high abundance in PLST sister (>20%). Number symbol indicates the highest abundance of *Archaea* domain in PLST.

Both domains, *Bacteria* (90.16%) and *Archaea* (9.84%), were present in the samples. PLS salivary microbiome presented different profiles at the phyla level, with higher proportions of Fusobacteria at the PLST sister and Firmicutes and Proteobacteria at the PLSTL1 sister and Bacteroidetes and Firmicutes at the PLSTL2, as shown in **Figure 3**. The heatmap (**Figure 4**) shows the genera relative abundances of prokaryotic taxa. *Bacteroidales* F0058 (25.88%) and *Fusobacterium* (34.64%) dominated the microbiome of the PLST sister. The PLST microbiome also presented high abundances of *Tannarella* (1.12%), *Treponema* (14%), *Campylobacter* (5.53%), and *Aggregatibacter* (4.61%). *Streptococcus* dominated the microbiome of the PLSTL1 sister, comprising 32% of the total microbiome, with *Haemophilus* and *Caldivirga* in a relative abundance higher than 10%, while the PLSTL2 had higher abundances of *Lactobacillus* (8%) and *Porphyromonas* (3%). The *Archaea* domain corresponded to 10 genus-level taxa, all from the phyla Crenoarchaeota and Halobacteriota. The genus *Caldivirga* (family Thermoproteaceae) and *Sulfophobococcus* (family Desulfurococcaceae) comprised the core microbiome of *Archaea* among PLS sisters. *Caldivirga*’s relative abundance was 0.8% in PLST, 13.27% in PLSTL1, and 6.15% in PLSTL2. *Sulfophobococcus*’ relative abundance was 4.45% in PLST, 0.95% in PLSTL1, and 0.15% in PLSTL2. The Venn diagram (**Figure 5**) shows that the PLSTL microbiomes shared higher ASVs than the PLST. A core microbiome could be identified, including the organisms *Prevotella*, *Fusobacterium* (high abundance in all samples, and dominant in the toothed sister), *Caldivirga* (family Thermoproteaceae, belonging to the *Archaea* domain, high abundance in PLSTL), *Streptococcus* (low abundance in PLST, 0.7%), *Oribacterium*, *Aggregatibacter*, *Dehalococcoidia* 1226B1H1-22-FL, *Haemophilus*, *Actinomyces*, *Campylobacter*, *Gemella*, *Sulfophobococcus* (*Archaea*), *Capnocytophaga* (ubiquity, but in low abundance), and *Peptococcus*.

## Discussion

The oral phenotype of the PLS individuals in this study followed the syndrome profile with bone loss and early edentulism for the oldest sisters (PLSTL1 and PLSTL2). All of them had palmoplantar hyperkeratosis as well. The PLST (younger sister) presented tooth mobility, bone loss, deep probing depth, inflamed gum, and feeding difficulty. The evaluation of the probing depth was complex in the PLST individual because of the spontaneous gingival bleeding, gingival hyperplasia, intense tooth mobility, and the young age of the patient. The attempts to treat periodontitis in PLS by conventional methods are frustrated in most cases, which was the case of these sisters. This study shows differences in the salivary microbiome between PLST and PLSTL (PLSTL1 and PLSTL2). For the first time, the high relative abundance of uncultivated bacteria *Bacteroidales* F0058, *Caldivirga*, and *Sulfobococcus* (the last two from the *Archaea* domain) were detected in PLS, which was possible due to the next-generation sequencing method.

The salivary characterization of the patients showed that the PLS patients have normal pH, buffering capacity, and reliability. The three PLS patients presented severe or moderate hyposalivation, and hyposialia was found in PLSTL1 and PLSTL2. This finding suggests hyposalivation as a possible characteristic of the syndrome and corroborates the study of Lundgren et al. (1996). More studies are necessary to confirm this hypothesis, as hyposalivation is associated with a higher risk of oral infections (Pedersen et al., 2018; Shimomura-Kuroki et al., 2020). Salivary hypofunction may also play a role in the diet change, resulting in malnutrition and or weight loss, affecting life quality (Pedersen et al., 2018). It is essential to highlight that saliva plays an essential role in maintaining a balanced microbiota (Pedersen et al., 2018) and promoting oral health (Kilian et al., 2016). Curiously, the level of salivary glucose was high in PLSTL1. The salivary microbiome was altered in the presence of high salivary glucose concentration, showing a decrease in bacterial load (Goodson et al., 2017). In the study of Goodson et al., the order of the microbiome reduction was in the direction of aciduric strength of bacterial species, with *Prevotella* spp. being more sensitive and *Streptococcus mutans* among the most resistant. The relative abundance of *Streptococcus*, and *Prevotella* in salivary samples of PLSTL1 and PLSTL2 followed a similar profile in our study. *Streptococcus* dominated the PLSTL1 microbiome (salivary glucose >10mg/dl) and *Prevotella* dominated the microbiome in PLSTL2 (salivary glucose, <2 mg/dl). A high abundance of *Streptococcus*, *Treponema*, and *Campylobacter* in dental biofilms of PLS individuals was described by Albandar et al. (2012), using 16S ribosomal DNA cloning and the Human Oral Microbe Identification Microarray (HOMIM). However, our results showed fewer *Streptococcus* in the PLST sample using an next-generation sequencing (NGS) method. Albandar et al. (2012) used a cloning method, which leads to loss of diversity, particularly for uncultured microorganisms. Due to the methodological differences, we could better describe the abundance of other genera that should be involved in the dysbiosis of PLS.

Despite the high relative abundance of acidogenic *Firmicutes*, the PLST1 and PLST2 saliva pH was neutral. The mean salivary amylase concentration in PLSTL2 samples was 16,016.74 ± 2,399.40 U/dl, while PLSTL1 and PLST amylase concentrations were approximately 11,000 U/dl. An interesting study demonstrated that the copy number of the salivary amylase gene AMY was correlated with oral and gut microbiome composition and function (Poole et al., 2019). Amylase is a crucial salivary enzyme that hydrolyzes alpha bonds of starch and glycogen, beginning starch degradation in the mouth. According to the authors, the microbiomes of AMY1 low-copy individuals (AMY1 L) had enhanced capacity to break down complex carbohydrates. AMY1 high-copy subjects (AMY1 H) had a higher abundance of salivary *Porphyromonas*. Their gut microbiota had an increased abundance of resistant starch-degrading microbes. The OTUs that significantly discriminated the AMY1 H and AMY1 L groups belonged to the genera *Prevotella* (AMY1 H), *Haemophilus* (AMY1 L), *Neisseria* (AMY1 L), and *Porphyromonas* (AMY1 H). In this study, PLSTL2 had a higher abundance of *Prevotella* (23.56%) and *Porphyromonas* (2.68%) than the other PLS sisters, which corroborates the AMY H hypothesis of Poole et al. (2019). *Neisseria* was found only in PLSTL1 samples (3.28%), and *Haemophilus* had a higher relative abundance in PLSTL1 (17.12%) than in the saliva of the other PLS sisters. The amylase activity and concentration do not seem to be affected by cathepsin C deletion in mice (CTSC^−/−^), neither cathepsin-C colocalized in the zymogen secretory compartments (John et al., 2019). Therefore, the high amylase concentration in PLSTL2 is probably an isolated observation with no relationship with a cathepsin C mutation in PLS but deserved analysis as it can affect the microbiome. It is essential to highlight the high abundance of *Granulicatella* and *Veillonella* at the PLSTL sisters. Those genera are part of the oral mucosa specialized microbiome. Comparing PLSTL sisters with PLST, we must consider that the difference observed in the microbiome of PLST saliva is strongly related to the presence of teeth surfaces as physical supports for supra and subgingival plaques attachment.

The salivary microbial changes after PLS patients losing their teeth are expected since, it represents a loss in bacteria adherence surface (Aas et al., 2005; Socransky and Haffajee, 2005; Gazdeck et al., 2019). According to Zaura et al. (2009), the communities obtained in saliva samples are closer to mucosa communities than dental sites. The teeth loss cause changes in the oral cavity that result in the loss of bacterial taxa in soft tissues (Gazdeck et al., 2019). The biofilm that formed on teeth develops quickly and has a more significant proportion of species than edentulous individuals (O’Donnell et al., 2015). The literature suggests the demand for hard surfaces for the colonization of some species and the gingival crevice fluid from gingival sulci or periodontal pockets for the colonization of others, which can explain the main differences found in the microbiome of the partially edentulous and edentulous PLS sisters. However, the edentulous subjects (PLSTL1 and PLSTL2) that wore full dentures at the baseline of this study (for almost 2 years and 6 months, respectively) also showed striking differences in their microbiome. Socransky and Haffajee (2005) showed that, in general, the microbial profile was more similar between healthy and periodontitis subjects than edentulous subjects. The data of Socransky and Haffajee study (2005) supported the concept that the nature of the hard tissue surface in the oral cavity impacts the nature of colonizing species on hard tissue surfaces and soft tissue surfaces. Individuals who were fully edentulous and had been wearing dentures for at least 1 year showed differences in their microbiome compared with those present in samples of supragingival plaque from subjects who were periodontally healthy or had chronic periodontitis. They corroborated the denture surfaces as support for recolonization by the abundance of *S. mutans* and lactobacilli in the mouths of edentulous individuals wearing dentures. Their data indicated that *Porphyromonas gingivalis*, *Aggregatibacter actinomycetemcomitans*, and *Tannerella forsythia* could be detected in edentulous subjects 1 year or longer after all teeth have been extracted. The environmental characteristics as a consequence of the high salivary glucose of PLSTL1, the severe hyposalivation and high amylase level of PLSTL2, and other factors like the time of dentures usage and oral hygiene profiles can be responsible for the differences found in PLSTL sisters microbiome.

The PLS individuals of this study had a genetic predisposition of ineffective cathepsin C that led to inflammation and stage IV periodontitis, which suits the inflammation-mediated plymicrobial-emergence and dysbiotic exacerbation (IMPEDE) model (Van Dyke et al., 2020). The periodontitis inflammation process can trigger dysbiosis, and the own dysbiosis enhances the inflammatory response (Van Dyke et al., 2020). The dysbiosis theory in periodontitis is related to the microbiological community’s transition from Gram-positive commensal to Gram-negative-enriched “inflammatogenic” community (Kirst et al., 2015). Interestingly, *Bacteroidales* F0058 (clone AU126, OT274, or HOT274), presumptively a Gram-negative bacterium, massively dominated the microbiome of the PLST sister as an exclusive ASV related to the presence of teeth, with no reads in PLSTL microbiomes. In the Human Microbiome Project (HMP) cohort of 210 adults, *Bacteroidales* F0058 [provisionally assigned *Bacteroidales Neisseriaceae* (G-1) *bacterium* HMT-274] was common in adult nostrils (Escapa et al., 2018). The investigation of Kumar et al. (2003) on potential periodontal pathogens in 66 individuals showed that *Bacteroidales bacterium* HMT-274 were among the most strongly associated with the formerly chronic periodontitis, comparable or more significant than species *P. gingivalis* and *T. forsythia*. The prevalence of *B. bacterium* HMT-274 was significantly high in the study population with periodontitis (82%). Li et al. (2006) also found a significantly high prevalence of *B. bacterium* HMT-274 in subgingival plaque in formerly chronic periodontitis (77.1%) and plaque-induced gingivitis (61.5%). *Bacteroidales bacterium* HMT-274 (F0058), as-yet-uncultivated bacterium, has no potential described growth partners. Escapa et al. (2018), in their ANCOM analysis, detected only the group-specific taxon and no other species with differential relative abundance related to *B. bacterium* HMT-274. However, according to Oliveira et al. (2016), the new species *B. bacterium* HMT-274 is not a consequence of periodontitis but is likely to play a crucial role in initiating the disease.

Another striking result of our study was the presence of the *Archaea* domain, corresponding to 24 ASVs and 10 genus-level taxons, all from the phylum *Crenoarchaeota* and *Halobacteriota*. The *Haloarcula* was recently found to be part of the gastrointestinal tract microbiome (Kim et al., 2020). Our study is the first report on the presence of high abundance of *Archaea* in the human salivary microbiome. Although mostly methanogen *Archaea* has been detected at the oral cavity so far (Belmok et al., 2020), and other signs of archaeal presence in oral samples were associated with samples contamination, the high abundance of *Caldivirga* and *Sulfophobococcus* represents evidence of the importance of those organisms (or their taxonomic-related), possibly associated with dysbiotic sites. *Crenarchaeota* was already detected in human fecal samples, suggesting their presence in the microbiota of the human digestive ecosystem (Rieu-Lesme et al., 2005). In the salivary samples of the PLS sisters, both genus *Caldivirga* (family Thermoproteaceae) and *Sulfophobococcus* (family Desulfurococcaceae) comprised the core microbiome, ranging from 0.8% to 13% of relative abundance. Some studies explained part of the indirect/direct role of archaea in inflammation in specific sites and the proinflammatory potential of some species (Borrel et al., 2020). When there is a dysbiosis or infection, various body sites are known to have a higher prevalence of archaea, especially skin and oral cavity. In severe periodontitis, it was shown that the shift to anaerobic fermentative bacteria is accompanied by an increase in *Archaea* (Dabdoub et al., 2016). The PLST individual presented over 5% relative abundance of *Crenarchaeota* (4.45% *Sulfophobococcus* and 0.8% *Caldivirga*). PLSTL1 and PLSTL2 presented 0.95% and 0.15% *of Sulfophobococcus*, while *Caldivirga* abundance was 13.27% and 6.15%, respectively. This result also suggests an archaeome-related dysbiosis in PLS that changes with edentulism. Pausan et al. (2019) discussed the importance of archaea-specific procedures, as universal approaches fail to picture the diversity of archaeal signatures in the previous analysis. Although their combination of universal primers 515F-806R showed coverage of 94.6% of archaea *in silico* (95.90% of Euryarchaeota, 94.60% of Thaumarchaeota, and 89.10% of Nanoarchaeota), the authors did not find similar detection levels experimentally. In their study, a nested PCR approach based on a first PCR with primer pair 344F-1041R, followed by a second PCR with 519F-806R, was superior for analyzing the archaeome of the gastrointestinal tract, oral cavity, and skin. In our study, the combination of universal primers 515F-806R showed a good coverage for the *Archaea* domain in the salivary samples of the PLS individuals, corroborating the *in silico* findings of Pausan et al. (2019). The high levels of archaea detection in our study may reflect a specific feature of the syndrome, probably revealed due to the advances in the archaeal sequences in databases. We believe that the oral archaeome will be easily characterized following technological advances in the future.

Dissimilatory and assimilatory sulfate reduction taxons dominated the *Bacteria* and *Archaea* core microbiome in PLS, and they were massively abundant in PLST (Kanehisa and Goto, 2000). *Desulfurococcales* and *Sulfophobococcus* are anaerobes acidophilic that ferment sugars and peptides, using organic compounds to reduce sulfur and generate hydrogen sulfide (H_2_S), organic acids, and alcohols (DasSarma et al., 2009). The H_2_S from *Archaea* metabolism may serve as an electron donor for a diversity of aerobic chemotrophic and anoxygenic microorganisms, forming microbiological communities in sulfidic habitats (Blohs et al., 2019). The sulfate-reducing bacteria tended to prevail in pockets with bleeding on probing, and their presence significantly correlated with pocket depth (Langendijk et al., 2000 and Dabdoub et al., 2016). Possibly, *Sulfophobococcus* and *Caldivirga* have a similar correlation in PLS and developed an essential role in coaggregation to form an aggressive subgingival plaque. *Archaea* may indirectly participate in periodontal disease by serving as a hydrogen sink, thereby facilitating the proliferation of pathogenic secondary fermenters to levels beyond the one in its absence (Dabdoub et al., 2016). Therefore, *Archaea* may have contributed to a favorable environment for the sulfate-reducing bacteria in PLST. *Staphylococcus aureus*, *Prevotella intermedia*, and *Fusobacterium nucleatum* are organic sulfate-reducing bacteria (Kushkevych et al., 2020). *Fusobacterium* dominated the PLST microbiome (34.64%), consistent with a sulfidic habitat and with the crucial role of this bacterium in oral biofilm structure and ecology. *Fusobacterium nucleatum*, for example, was found to act as a bridge between early and late periodontal biofilms colonizers (Thurnheer et al., 2019). Moreover, *F. nucleatum* triggers the production of matrix metalloproteinases by the host and has enhanced hemolytic activity, and the production of H_2_S is a key virulence trait of this bacteria in periodontitis (Thurnheer et al., 2019). Members of the genus *Treponema* are also capable of homoacetogenesis (hydrogen-consuming process), including the periodontitis pathogen, *Treponema denticola*. PLST presented high abundances of *Treponema* (14%), *Tannarella* (1.12%), and *Campylobacter* (5.53%), frequently enriched in the periodontitis-associated microbiome (Socransky and Haffajee, 2005, Hajishengallis et al., 2012). Those results support the hypothesis of possible syntrophic interactions between *Archaea, Treponema*, and other members of the red complex (Dabdoub et al., 2016), in a highly “inflammatogenic” subgingival community.

**Figure 6:**
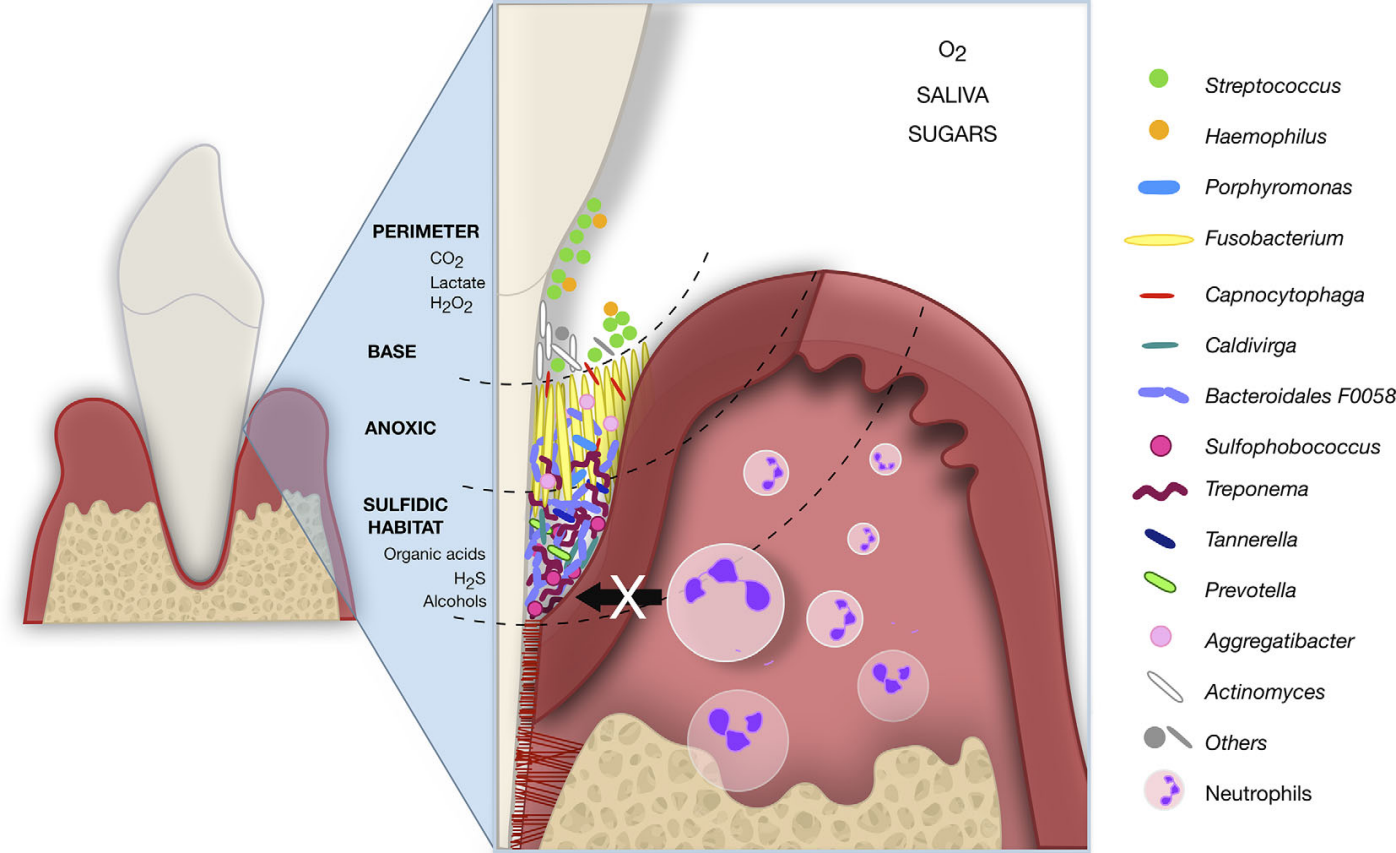
Hypothetical subgingival microbiome biogeography of PLST. The just erupted teeth serve as attachment support for ubiquitous commensals that form an initial pellicle (*Streptococcus* and *Actinomyces*). The neutrophils infiltration and first line of defense are impaired due to an ineffective cathepsin C. The attached aerobic microbes then serve as a substrate for further colonizers. *Fusobacterium*, also a ubiquitous bacterium, plays a crucial role as a bridge between the first and late colonizers. *Fusobacterium* produces hydrogen sulfate (H_2_S), overgrows, and creates a sulfidic habitat ideal for anaerobic such as *Treponema*, *Porphyromonas*, *Prevotella*, *Tannerella*, *Sulfophobococcus*, *Caldivirga*, and *Bacteroidales* F0058. The schematic image represents a hypothesis for PLST and was inspired by Mark Welch et al. (2016).

Over the years, the microbiota and host response interactions as the initial causal agent of periodontitis became more evident. However, the pathological shift from localized and contained to progressive and destructive periodontitis has not been clarified yet (Bartold and Van Dyke, 2019; Van Dyke et al., 2020). The development of aggressive periodontium destruction in PLS individuals may be a model to understand how genetics, cytokines, immunological factors, and microbiome interact for the periodontitis outcomes. In PLS, the genetic disorder of cathepsin C (CTSC) came first and led to dysbiosis in the individuals of this study. Taken together the previous literature and our findings, we hypothesized a subgingival microbiome biogeography in PLST that begins with the teeth eruption (**Figure 6**). As soon as the teeth begin to erupt in the dental arch of PLST individual, the salivary pellicle covers them providing attachment support and substrate for the ubiquitous commensal microorganisms. Thereafter, some microorganisms comprise the biofilm’s biogeography on a micrometer scale, as proposed by Mark Welch et al. (2016); *Streptococcus*, *Haemophilus*, *Actinomyces*, *Capnocytophaga*, and *Fusobacterium* can colonize the tooth surface (**Figure 6**). The impaired immune system cannot provide the first line of cellular defense due to an ineffective cathepsin C. Neutrophils elastase (NE) is not active or reduced, making the infiltration of neutrophils to the adjacent tissues impossible, impairing phagocytosis, cytonemes, and NETs formation (John et al., 2019). The lack of neutrophil’s defense can reduce the control of the microbial load, allowing a massive biofilm accumulation (**Figure 6**). The first colonizers proliferate, decreasing the level of O_2_. The environment is highly favorable to other microorganisms coadhere or coaggregate, shifting from Gram-positive to Gram-negative anaerobes (Kirst et al., 2015). For instance, *Fusobacterium*, which abundantly dominates the microbiome of PLST, creates an anoxic lay and dictates the biofilm structure and ecology (Mark Welch et al., 2016). *Fusobacterium nucleatum*, for example, overgrew in a sulfidic environment and showed to be a high producer of hydrogen sulfide (H_2_S) (Thurnheer et al., 2019). The same characteristic applies to another producer of H_2_S, the archaea *Sulfophobococcus* (Blohs et al., 2019). Anaerobes that use sulfate as a terminal electron acceptor for anaerobiosis in the dissimilatory reduction process of sulfur (DRS) and organic compounds as source of energy find a perfect sulfidic habitat (Thurnheer et al., 2019; Kushkevych et al., 2020). The environment conditions, the synergy and microbial competition together, can favor the proliferation of other Gram-negative and anaerobic microorganisms such as *Archaea*, *Treponema*, *Bacteroidales* F0058, *Tannerella*, and *Porphyromonas* mimicking a formal aggressive periodontitis consortium in PLST (**Figure 6**).

This study suggests that host response and the resident microbiome are linked to a bidirectional imbalance between health and disease in PLS. The PLST individual does have a microbiota different from that of the periodontitis’s aggressiveness previously recognized. A microbiome possibly favored by a sulfidic habitat, capable of resisting conventional periodontal treatment and antibiotic therapy. For instance, archaea are recognized as resistant to antimicrobial agents that interfere with peptidoglycan biosynthesis since their cell wall lacks peptidoglycan. The three PLS sisters were provided with intensive periodontal treatment, mechanical therapy, and oral hygiene instructions, including methods for denture cleaning before and after the study. The attempts of treatment prior to the study were unsuccessful, even conventional antibiotic therapy. All consequences of an environment where neutrophils and their frontline defensive mechanisms are inexistent or substantially reduced. The phenotype of Papillon–Lefèvre Syndrome highlights the microbiome dysbiosis and the fundamental role that neutrophils play in maintaining oral and skin health. Considering the high relative abundance in PLST, the genus *Fusobacterium*, *Treponema*, *Tannerella*, and *Sulfophobococcus* are possible candidates to form a consortium with *Bacteroidales* F0058; this is a hypothesis that deserves future investigation. Undeniable, the archaeome is ubiquitous in the salivary microbiome and more complex than previously thought. The knowledge about the PLS microbiome is exceptionally relevant in the search for specific successful treatments. New broad-spectrum antimicrobial agents and second-generation retinoid acitretin (vitamin A derivative) are potential alternatives to provide a better prognosis for the outcomes of PLS.

## Data Availability Statement

The datasets presented in this study can be found in online repositories. The names of the repository/repositories and accession number(s) can be found in the article.

## Ethics Statement

The studies involving human participants were reviewed and approved by Ethics Committee of the Faculty of Health Sciences of the University of Brasília (FS-UnB; no. 2.974.167). Written informed consent to participate in this study was provided by the participants’ legal guardian/next of kin. Written informed consent was obtained from the individual(s), and minor(s)’ legal guardian/next of kin, for the publication of any potentially identifiable images or data included in this article.

## Author Contributions

GL conceived ideas for this manuscript and was involved in data interpretation and manuscript drafting. LMS conducted experiments. GL was involved in the patient’s diagnosis, treatment, and manuscript drafting. LB conducted experiments and taxonomy assessment. LM conducted experiments and taxonomy assessment. LO was involved in study design, experiments, and manuscript drafting. ND-T conceived ideas for this manuscript and was involved in study design, data interpretation, and manuscript drafting. LPS conceived ideas for this manuscript and was involved in study design, experiments, data interpretation, and manuscript drafting. All authors contributed to the article and approved the submitted version.

## Funding

The study was supported by Institutional funds from the University of Brasília (PROAP-DGP/UnB No. 02/2019).

## Conflict of Interest

The authors declare that the research was conducted in the absence of any commercial or financial relationships that could be construed as a potential conflict of interest.

## Publisher’s Note

All claims expressed in this article are solely those of the authors and do not necessarily represent those of their affiliated organizations, or those of the publisher, the editors and the reviewers. Any product that may be evaluated in this article, or claim that may be made by its manufacturer, is not guaranteed or endorsed by the publisher.
